# Sequential colorectal cancer–based therapy including anti-angiogenics and 3^rd^-line trifluridine/tipiracil-bevacizumab for metastatic urachal adenocarcinoma: a case report

**DOI:** 10.3389/fonc.2026.1933091

**Published:** 2026-10-29

**Authors:** Shahla Abu Naib, Marco Gerlinger

**Affiliations:** 1 Leaders in Oncology Care, HCA Healthcare UK, London, United Kingdom; 2 Barts Cancer Institute, Queen Mary University of London, London, United Kingdom

**Keywords:** anti-VEGF therapy, bevacizumab, case report, trifluridine/tipiracil, urachal carcinoma

## Abstract

Urachal adenocarcinoma is a rare malignancy without prospective treatment trials. Management of metastatic disease is typically extrapolated from colorectal cancer (CRC) due to histologic and molecular similarities. An 80-year-old woman with metastatic urachal adenocarcinoma received sequential CRC-based therapy including first-line CAPOX plus bevacizumab, second-line FOLFIRI plus bevacizumab, third-line trifluridine/tipiracil plus bevacizumab, in addition to brief administration of regorafenib and fruquintinib in the fourth- and fifth-line setting. Oxaliplatin was discontinued after one cycle due to worsening of vision on a background of pre-existing retinopathy. The longest disease control (time to progression ~11 months) occurred with trifluridine/tipiracil plus bevacizumab. To our knowledge, this represents the first report describing use of this combination in the third-line setting for metastatic urachal carcinoma. Overall survival was 27 months. This case is hypothesis-generating for the use of modern CRC-based therapies, including anti-VEGF–containing combinations.

## Introduction

Urachal adenocarcinoma is an exceptionally rare malignancy with an incidence of ~1 case per 5 million population annually, accounting for <1% of bladder cancers (1–3). Most tumours arise from the urachal remnant at the bladder dome and display enteric differentiation (1, 4). Immunohistochemical and molecular analyses frequently demonstrate CK20 and CDX2 positivity and recurrent alterations in KRAS, APC and TP53, closely resembling colorectal adenocarcinoma (4–6).

Due to its rarity, no prospective randomised trials have been undertaken. Treatment recommendations are largely extrapolated from metastatic colorectal cancer (mCRC) based on retrospective case series and consensus guidance (7–9). The only formally consensus-recommended regimen for advanced urachal carcinoma is FOLFOX, with FOLFIRI also reported in case series. Notably, the role of bevacizumab is not addressed in existing guidelines (7). More recently, the refractory mCRC armamentarium has expanded to include trifluridine/tipiracil plus bevacizumab (10) and fruquintinib (11), agents for which no urachal-specific evidence existed at the time of this case, and which this report directly addresses.

We report a case of metastatic urachal adenocarcinoma treated sequentially according to a colorectal paradigm, demonstrating prolonged survival with detailed correlation of tumour marker kinetics and radiologic response.

## Case presentation

An 80-year-old woman presented in May 2022 with abdominal pain and haematuria. Biopsy of a bladder dome lesion which expanded to the urachus demonstrated a moderately differentiated adenocarcinoma, positive for CK20 and CDX2, with only scanty single-cell staining for GATA3, CK7 and the high-molecular-weight cytokeratin 34βE12, arguing against a urothelial origin. β-catenin showed diffuse membranous positivity with negative nuclear staining, excluding a Wnt-pathway-driven mimicker. This immunophenotype, together with the site of origin at the bladder dome extending into the urachal remnant and exclusion of a colorectal primary by colonoscopy, was considered diagnostic of urachal adenocarcinoma of enteric subtype. PET-CT (**Figure 1**) revealed widespread peritoneal and omental metastases with likely pulmonary involvement, corresponding to Sheldon/Mayo clinical stage IVB (distant metastatic) disease at presentation (9). No WHO/International Society of Urological Pathology (ISUP) staging system exists for urachal carcinoma; the Sheldon/Mayo system applied here remains the field-standard clinical staging classification for this rare malignancy. In retrospect, a lesion identified on imaging that was performed one year earlier to investigate recurrent urinary tract infections had been interpreted as a bladder hernia; this is now considered likely to have represented the primary urachal tumour. A colorectal malignancy was excluded by colonoscopy. Performance status (PS) was 0. She had a background of significant PRPH2-associated retinopathy with severely reduced visual acuity bilaterally. Molecular profiling identified a *KRAS G12V* mutation as well as *APC*, *FBXW7* and *TP53* mutations, with a microsatellite-stable (MSS) profile. Tumour mutational burden was low (1 mut/Mb) and the PD-L1 combined positive score was 5. The microsatellite-stable profile and low tumour mutational burden argued against benefit from immune checkpoint inhibition, and the KRAS G12V mutation precluded consideration of anti-EGFR therapy (cetuximab/panitumumab); together, these findings supported the colorectal cancer-based cytotoxic approach adopted. Pre-treatment tumour markers (TMs) in July 2022 were CEA 49 ng/mL (upper limit of normal <3.9 ng/mL) and CA19-9 123 kU/L (upper limit of normal < 35 kU/L).

**Figure 1:**
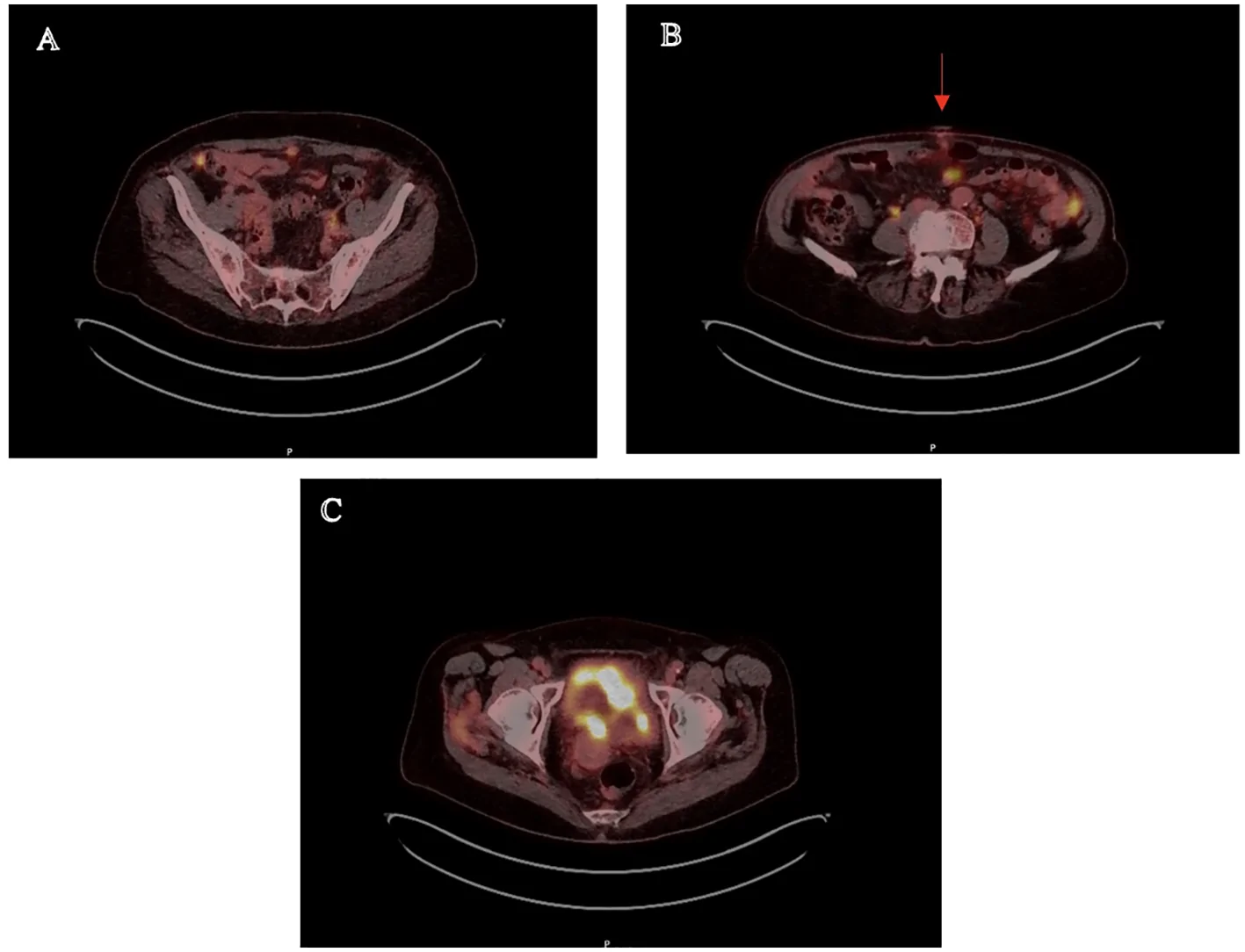
FDG PET-CT images illustrating the extent of the disease at the time of diagnosis: multifocal hypermetabolic peritoneal and omental nodules **(A, B)**, and an umbilical Sister Mary Joseph nodule (red arrow, **(B)**) and the urachal primary at the bladder dome **(C)**.

### First-line therapy

The multidisciplinary team recommended treatment according to a metastatic colorectal cancer paradigm. CAPOX was initiated mid-July 2022, consistent with mCRC practice, where fluoropyrimidine and oxaliplatin combination chemotherapy constitutes a standard first-line therapy (12).

After one cycle, oxaliplatin was discontinued due to acute peripheral visual impairment in the context of pre-existing PRPH2-associated retinal dystrophy. Ophthalmological review confirmed no new structural retinal changes on imaging, and the deterioration was attributed to oxaliplatin neurotoxicity superimposed on this underlying retinopathy. Oxaliplatin is recognised to cause visual field disturbances as a rare adverse effect (≥1/10,000, ≤1/1,000) (13). First-line treatment therefore followed a CAPOX ×1 → capecitabine alone ×2 → capecitabine plus bevacizumab ×6 sequence: oxaliplatin (130 mg/m², standard full dose), capecitabine monotherapy (1000 mg/m² twice daily, days 1–14, 21-day cycles) was continued alone for cycles 2 and 3. Capecitabine required a dose reduction to 80% from cycle 3 after an infective episode, maintained until escalation back to 100% at cycle 9 in response to rising TMs. Bevacizumab (7.5 mg/kg every 3 weeks) was added from cycle 4 and continued through cycle 9, the final cycle of first-line treatment, giving six cycles of capecitabine plus bevacizumab in total. No GCSF was required during first-line chemotherapy.

TMs declined substantially to a nadir of CEA 26 ng/mL and CA19-9 61 kU/L (**Figure 2**). CT at 3 months demonstrated stable disease by size criteria but appreciable cavitation of pulmonary metastases, interpreted as a favourable response. By 6 months, markers had risen to CEA 72 ng/mL and CA19-9 75 kU/L; a contemporaneous CT confirmed progression of the omental disease.

**Figure 2:**
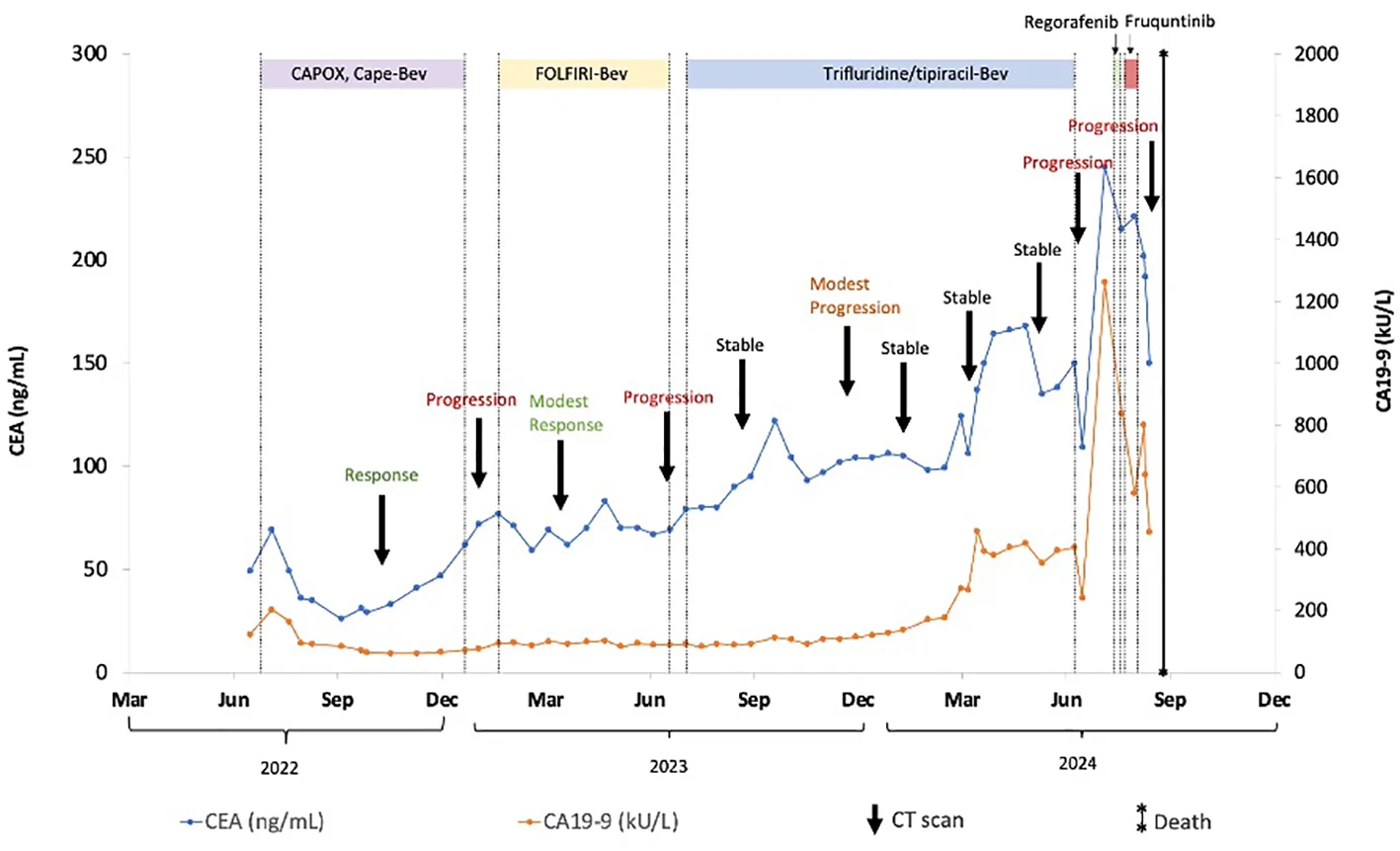
Longitudinal tumour marker kinetics (CEA and CA19-9) with integrated treatment timeline and CT timepoints across 27 months. Radiologic interpretation detailed in text.

### Second-line therapy

She commenced FOLFIRI plus bevacizumab in February 2023, in keeping with colorectal sequencing practice: irinotecan (standard dose 180 mg/m²) was reduced to 50% (90 mg/m²) for cycle 1, together with calcium folinate 350 mg and infusional fluorouracil 2400 mg/m² over 46 hours, in a 14-day cycle. Bevacizumab (5 mg/kg) was continued given its established survival benefit beyond first progression in mCRC (10, 12, 14). The 50% starting dose of irinotecan was chosen because, although irinotecan does not share oxaliplatin’s neurotoxicity profile, there was very little data on whether irinotecan could affect her rare macular dystrophy; a cautious starting dose allowed any unexpected adverse effects to be assessed carefully before escalation. Pegfilgrastim was administered from cycle 1; irinotecan was escalated to 70% (126 mg/m²) from cycle 7.

CT at 6 weeks showed marginal improvement with reduction in primary tumour and omental disease. TMs initially showed a modest reduction (**Figure 2**). CT at approximately 5 months confirmed radiological progression with increased pulmonary and peritoneal disease; TMs showed a modest increase at that point (CEA 69 ng/mL; CA19-9 90 kU/L).

### Third-line therapy

Following progression on FOLFIRI plus bevacizumab, treatment was switched to trifluridine/tipiracil plus bevacizumab in July 2023. Trifluridine/tipiracil has demonstrated survival benefit in refractory mCRC (15), and the SUNLIGHT trial established improved outcomes with bevacizumab addition (16). The regimen was delivered at the standard full dose (trifluridine/tipiracil 35 mg/m² twice daily on days 1–5 and 8–12, plus bevacizumab 5 mg/kg on days 1 and 15, of a 28-day cycle) throughout 11 cycles, with pegfilgrastim added from cycle 3.

No dose reductions were required during the 11 cycles of third-line therapy. Pegfilgrastim was introduced from cycle 3 as primary prophylaxis to maintain dose intensity, in the context of the cumulative myelosuppressive burden of prior treatment lines and an observed downward neutrophil trend, rather than in response to a specific neutropenic complication necessitating dose reduction.

A restaging CT at 6 weeks demonstrated stable disease. Imaging at 5 months showed a slight increase in the size of the primary tumour, as well as the lung and peritoneal metastases. As the TM were relatively stable, a decision was made to perform a repeat CT at short interval after another 6 weeks and this demonstrated a slight peritoneal reduction with stable pulmonary disease. This reassessment-rather-than-discontinuation approach reflected routine multidisciplinary practice in the absence of formal RECIST-defined progression at the 5-month timepoint. Scans at 8 and 10 months confirmed ongoing stability; imaging at 11 months showed increasing ascites, representing the definitive progression event. As no formal RECIST v1.1 response assessment was performed, this interval is best characterised as time to progression (TTP) under routine clinical evaluation, calculated from treatment initiation to the multidisciplinary determination of progression based on worsening ascites, rather than as a protocol-defined progression-free survival or response duration. This regimen achieved the longest disease control, for approximately 11 months. An initial transient TM spike at 2 months was followed by a sustained plateau through late 2023.

From approximately 8 months, a clear rise in TMs became evident while CT at that point still confirmed stable disease. Biochemical progression thus preceded radiological progression by several months.

### Fourth-line therapy

Following progression on trifluridine/tipiracil, subsequent VEGFR-targeted therapy was attempted. Regorafenib, a multikinase inhibitor approved in refractory mCRC (17), was commenced in July 2024 at a reduced starting dose of 80mg/day (50% of the standard dose of 160mg/day). Regorafenib was discontinue after only one week due to significant fatigue and generalized myalgia.

### Fifth-line therapy

Fruquintinib, a selective VEGFR inhibitor with demonstrated survival benefit in refractory mCRC (FRESCO and FRESCO-2 trials) (11), was initiated in July 2024 at a reduced dose of 3 mg/day (standard dose: 5 mg/day). The TM decline which has started with regorafenib treatment continued with fruquintinib exposure. However, fruquintinib had to be held after 13 days of treatment due to a decline in the patient’s performance status. Due to the brevity of the exposure to both agents, it is not possible to definitively assess their activity.

### Terminal course

In mid-August 2024, the patient was admitted with delirium, electrolyte derangement, ascites and constipation. She was treated for a new urinary tract infection and required ascitic drainage. She subsequently developed abdominal pain and a CT scan demonstrated worsening multifocal peritoneal disease with small bowel obstruction. She underwent an emergency laparotomy with formation of a defunctioning ileostomy. She was discharged to her home with community palliative care support and died at the end of August 2024 due to disease progression, 27 months after diagnosis.

A summary of systemic therapy sequencing, radiologic response across the 27-month disease course is provided in **Table 1**.

**Table 1:** Sequential systemic therapy with integrated radiologic response in metastatic urachal adenocarcinoma.

Line	Regimen	Time to progression	Best radiologic response
Baseline	*—*	*—*	Metastatic deposits in the peritoneum and lungs
First-line	CAPOX ×1 → Capecitabine alone ×2 → Capecitabine + Bevacizumab ×6 (9 cycles total)	~6 months	Stable with cavitation of pulmonary metastases (favourable response, ~3 months); marginal progression (~6 months)
Second-line	FOLFIRI + Bevacizumab (11 cycles)	~5 months	Marginal improvement (~6 weeks); radiological progression (~5 months)
Third-line	Trifluridine/tipiracil + Bevacizumab (11 cycles)	~11 months	Stable to ~10 months; increasing ascites at ~11 months
Fourth-line	Regorafenib (80 mg/day)	~1 week	*—*
Fifth-line	Fruquintinib (3 mg/day)	~2 weeks	*—*
Outcome	*—*	OS 27 months	

OS, overall survival. Radiologic response and time to progression reflect routine clinical CT interpretation; formal RECIST v1.1 review was not performed (see Limitations).

## Discussion

This case illustrates three notable aspects of a CRC-based treatment approach in metastatic urachal adenocarcinoma.

First, sequential CRC-based systemic therapy that included the VEGF antibody bevacizumab produced meaningful disease control across three lines, resulting in an overall survival of 27 months, which substantially exceeds the reported median survival of approximately 12 months for metastatic urachal carcinoma in historical series (9). This comparison should be interpreted cautiously given differences in patient selection, baseline performance status, disease burden, and treatment availability across historical cohorts and the present case; the patient’s excellent baseline performance status (ECOG PS 0) despite her advanced age (80 years) may have been an important contributor to her ability to tolerate sequential multi-line CRC-based therapy. Fourth- and fifth-line exposure to regorafenib and fruquintinib was brief and occurred in the context of rapid clinical deterioration, precluding meaningful assessment of activity. The patient derived benefit from capecitabine and bevacizumab therapy (approximately 6 months). The prolonged disease control is consistent with meaningful activity of this well-tolerated combination, although the independent contribution of bevacizumab cannot be distinguished from that of capecitabine in this setting.

Irinotecan, 5FU and bevacizumab subsequently controlled the cancer for approximately 5 months. each combined with bevacizumab. The prolonged disease stabilization achieved with third-line trifluridine/tipiracil plus bevacizumab (time to progression of approximately 11 months) was particularly notable, exceeding the median progression-free survival reported for this regimen in pre-treated mCRC clinical trial populations (10, 15, 16). These observations are hypothesis-generating for the use of a colorectal cancer therapy sequence, including anti-angiogenic drugs, in metastatic urachal cancer, though a single case cannot establish efficacy. As bevacizumab was co-administered with cytotoxic chemotherapy across all three treatment lines, its independent contribution to the disease control observed cannot be determined from this case, and these findings should not be interpreted as evidence of a survival benefit specifically attributable to VEGF inhibition in urachal carcinoma.

Second, at the time trifluridine/tipiracil plus bevacizumab was initiated, no published data described its use in urachal carcinoma. Only recently has a single case report described this combination in metastatic urachal carcinoma in the second-line setting (18). In that report, a 53-year-old man received trifluridine/tipiracil plus bevacizumab as second-line therapy after ten cycles of FOLFOX, achieving a progression-free survival of 17 months, although bevacizumab was switched to monotherapy after six cycles because of hematological toxicity. Our case extends this observation to a third-line, more heavily pre-treated 80-year-old patient, in whom a shorter but still clinically meaningful time to progression of approximately 11 months was achieved with the full combination maintained throughout, suggesting that activity may be retained even after two further lines of CRC-based therapy and in an older patient. Together, these observations are hypothesis-generating for potential activity of VEGF-directed strategies in urachal adenocarcinoma and warrant confirmation in larger series.

Third, CEA and CA19–9 broadly reflected the overall disease trajectory, with markers declining during first-line therapy and rising sharply in the terminal phase. However, correlation with individual radiological assessments was imperfect. During second-line FOLFIRI plus bevacizumab, CT showed marginal improvement at 6 weeks while markers fluctuated without declining, and no meaningful marker rise accompanied CT-confirmed progression. During third-line therapy, sharp biochemical progression preceded CT-confirmed progression by several months, demonstrating that the two modalities provide complementary information. Although TMs are not formally validated in urachal carcinoma, in this patient their kinetics may have provided complementary information to imaging.

## Strengths and novelties

The principal novelty of this report is the description of third-line trifluridine/tipiracil plus bevacizumab in metastatic urachal carcinoma, a setting for which no prior evidence existed at the time of treatment and for which, to our knowledge, only one other case has since been published, in the second-line setting after a single prior line of chemotherapy, compared with two prior lines of CRC-based therapy in the present case (see Discussion). The granularity of longitudinal tumour marker and radiologic data across a 27-month disease course, permitting direct comparison between biochemical and radiological progression, is a further strength, along with the demonstration of multi-line disease control exceeding historical median survival for this rare malignancy.

## Limitations

This report has several limitations. As a single-patient case report, the observed benefit of CRC-based, anti-angiogenic-containing therapy cannot be generalized, and the contribution of individual agents versus the overall sequencing strategy cannot be disentangled. Radiological response was assessed qualitatively by the treating radiologist and oncologist as part of routine clinical care; formal retrospective RECIST v1.1 review could not be performed in this routine clinical setting, and the treatment durations and response descriptors reported here therefore reflect routine clinical interpretation rather than protocol-defined response criteria. Tumour marker kinetics, while informative here, are not formally validated in urachal carcinoma and were interpreted alongside imaging rather than as a standalone measure. Brief fourth- and fifth-line exposure to regorafenib and fruquintinib, curtailed by toxicity and clinical deterioration, precludes any meaningful assessment of activity for these agents in this setting.

## Conclusion

This case indicates that metastatic urachal adenocarcinoma patients can derive meaningful benefit from modern CRC-based systemic therapy. CRC-guided treatment sequencing achieved multi-line disease control over 27 months, with third-line trifluridine/tipiracil plus bevacizumab providing the longest period of stabilization. In the absence of prospective trials, carefully documented real-world experience remains essential to inform management of this rare malignancy.

## Data Availability

The original contributions presented in the study are included in the article/supplementary material. Further inquiries can be directed to the corresponding author.
